# Patriarchy at the helm of gender-based violence during COVID-19

**DOI:** 10.3934/publichealth.2021003

**Published:** 2020-12-21

**Authors:** Sumbal Javed, Vijay Kumar Chattu

**Affiliations:** 1 School of Public Health, The University of Hong Kong, Hong Kong; 2 Department of Medicine, Faculty of Medicine, University of Toronto, Toronto, ON M5G 2C4, Canada; 3 Global Institute of Public Health, Thiruvananthapuram, Kerala, India

**Keywords:** gender-based violence, COVID-19, equity, sexual and reproductive health, human rights, Ebola, pregnancy

## Abstract

Gender-based violence (GBV) or violence against women and girls (VAWG), is a global pandemic that affects 1 in 3 women in their lifetime and VAWG is one of the most prevalent human rights violations in the world. The high level of investment going into the COVID-19 recovery plan is a unique opportunity to reshape our patriarchal society, to coordinate across sectors and institutions and to take measures to reduce gender inequalities. Relief efforts to combat the pandemic should take the needs of the vulnerable population, particularly women and girls afflicted by GBV into consideration, as their needs were mostly ignored in the recovery plan of Ebola. GBV is linked to dominance, power and abuse of authority or because any calamity, be it a pandemic, conflict or a disaster. This will further exacerbate pre-existing gendered structural inequalities and power hierarchies as protective mechanisms fail leaves women and girls more vulnerable, fueling impunity for the perpetrators. There is a need for international and domestic violence prevention policies to not only focus on narrowly defined economic or political ‘empowerment’ because that is insufficient when it comes to challenging existing gender inequalities. Incorporating an individual's religious beliefs and community of faith (mosque, church, temple or synagogue) can offer a support system for an individual and her/his family amid a public health crisis. There is a need to engage men and boys by tailoring messages to challenge gender stereotypes and unequal gender roles to overcome patriarchy.

## 1. Introduction

Gender-based violence (GBV) or violence against women and girls (VAWG), is a global pandemic that affects 1 in 3 women in their lifetime [1] and VAWG is one of the most prevalent human rights violations in the world [2]. The high level of investment going into the COVID-19 recovery plan is a unique opportunity to reshape our patriarchal society, to coordinate across sectors and institutions and to take measures to reduce gender inequalities. Relief efforts to combat the pandemic should take the needs of the vulnerable population, particularly women and girls afflicted by GBV into consideration, as their needs were mostly ignored in the recovery plan of Ebola.

An increase in GBV during COVID-19 is nothing new; previously, during the Ebola crisis in West Africa, Guinea had reported a 4.5% increase in GBV and twice as many rapes. At the same time, Sierra Leone and Liberia saw a rise in teenage pregnancy rates [3].

As the GBV incidences increase during COVID-19, recourses for survivors are under strain, one-third of women reported difficulty in accessing resources. While shelter-in-place restrictions are in place, it is estimated that additionally, GBV 15 million incidences will occur globally every three months. GBV is associated with higher mortality and morbidity for co-occurring diseases [4].

Unfortunately, when the current pandemic ends it will not be the end of GBV. Beyond its direct impact on physical, mental health, this shadow pandemic will a have a wide-scale, long-standing and generational impact, for instance when a child is exposed to domestic violence, he/she is also at risk of being abused. Moreover, they may learn to tolerate and possibly inflict violence on others, further driving the intergenerational violence [5]. Child marriage is patriarchal disempowerment, and its practice widely recognized as a violation of girls' human rights [6]. The United Nations Population Fund has predicted that in the next ten years, additional 13 million child marriages will take place globally, which could have been prevented if not for the disruption caused by the COVID-19 [7].

Sexual and gender-based violence (SGBV) refers to any act that is perpetrated against a person's will and is based on gender norms and unequal power relationships. It includes threats of violence and coercion, which can be physical, emotional, psychological, or sexual and can take the form of a denial of resources or access to services [5].

Before COVID-19 came along, GBV was already one of the most significant human rights violations. In patriarchal societies, women internalize GBV. They are unable to perceive their husband's abusive behavior as a violation; instead, they believe that he is exercising a right that serves her interest [8]. GBV is linked to dominance, power and abuse of authority and because of any calamity, be it a pandemic, conflict or a disaster will exacerbate pre-existing gendered structural inequalities and power hierarchies as protective mechanisms fail leaves women and girls more vulnerable, fueling impunity for the perpetrators.

Power and decision-making rights give greater access to, and control over resources usually makes men more powerful than women. This power and control over resources and decisions are institutionalized through the laws, policies and regulations of formal social institutions [9],[10]. VAWG represents a key priority in achieving gender equality around the world [11]. According to The UN chief António Guterres, the COVID-19 pandemic has illustrated “what we all know”, years of patriarchy has led to a male-dominated world and a male-dominated culture which has damaged everyone. He further added, “It is clear that we cannot go back to the failed policies that have resulted in the fragility we see around us—in healthcare systems, in social protection, in access to justice. This is the time to rebuild equal, inclusive, and resilient societies” [12]. We need to make sure that we emerge from this once-in-a-generation crisis with women as a powerful force at the center of recovery.

## 2. Conclusions

Patriarchy perpetuates that girl and women are of less value, and in a patriarchal society, GBV often is widely accepted by society [13]. Currently, 1 in 4 countries has no laws that specifically protect women from domestic violence [14]. Explicit recognition of GBV is essential when developing health care policies, laws and legislations because what is unnamed is more likely to be unsupported. Studies have shown that commonly used measures of gender quality scores, such as female labor force and the number of seats held by women in national parliament did not significantly influence society's acceptance of GBV. There is a need for international and domestic violence prevention policies to not only focus on narrowly defined economic or political ‘empowerment’ because that is insufficient when it comes to challenging existing gender inequalities [8].

Patriarchal religious systems, pervasive behaviors and ideologies often negatively impact the lives of women, and many gendered health disparities can be attributable to it [4],[11]. Most of the countries around the world practice some form of faith, including the Arabian Peninsula. Hence, religious issues or concerns are likely to surface when seeking help or forming policies to eliminate GBV. These issues need to be addressed as they may become roadblocks and could jeopardize resolving gender issues. Therefore, it is imperative to develop an understanding of both secular and religious narratives to address patriarchy and its impacts, particularly GBV. Besides, incorporating an individual's religious beliefs and community of faith (mosque, church, temple or synagogue) can offer a support system for an individual and her/his family amid crisis [15].

COVID-19 response requires a paradigm shift in promoting policies and interventions that account for the varying impact of a pandemic on different genders—fostering inclusion of women's organizations, to protect and promote human rights throughout COVID-19 response plan. Ensuring that the emergency COVID-19 related regulations and policies uphold international human rights standards and considering gender in post-pandemic when allocating recourses and setting agendas [4],[7],[10].

Any efforts made to alleviate gendered health disparities must address any underlying social structures and norms. In addition to strengthening our healthcare systems for future emergencies, we need to improve our capacity to respond to current and future GBV by incorporating GBV protection policies into emergency response planning. There is a need to engage men and boys by tailoring messages to challenge gender stereotypes and unequal gender roles to overcome patriarchy.
